# Integrated tumor genomic and immune microenvironment analysis identifies predictive biomarkers associated with the efficacy of neoadjuvant therapy for triple‐negative breast cancer

**DOI:** 10.1002/cam4.5372

**Published:** 2022-10-21

**Authors:** Yanhui Zhu, Hongfei Zhang, Chaohu Pan, Gao He, Xiaoli Cui, Xiafei Yu, Xiaoqiang Zhang, Dongfang Wu, Junzhe Yang, Xian Wu, Haitao Luo, Xiaoan Liu

**Affiliations:** ^1^ Department of Breast Surgery The First Affiliated Hospital of Nanjing Medical University Nanjing Jiangsu China; ^2^ The First Affiliated Hospital Jinan University Guangzhou Guangdong China; ^3^ Department of Medicine YuceBio Technology Co., Ltd Shenzhen Guangdong China

**Keywords:** biomarkers, breast cancer, CD8 T cells, M2 macrophages, neoadjuvant chemotherapy therapy, TMB, TNBC

## Abstract

**Background:**

Although neoadjuvant chemotherapy (NAC) is currently the best therapy for triple‐negative breast cancer (TNBC), resistance still occurs in a considerable proportion, thus it is crucial to understand resistance mechanisms and identify predictive biomarkers for patients selection.

**Methods:**

Biopsy samples were collected from 21 patients with TNBC who underwent NAC. Whole‐exome sequencing (WES), targeted sequencing, and multiplex immunohistochemistry (mIHC) were carried out on the clinical samples and used to identify and validate potential biomarkers associated with response to NAC. In addition, data on 190 TNBC patients who had undergone chemotherapy were obtained from The Cancer Genome Atlas (TCGA) and analyzed to further validate our findings.

**Results:**

Both the tumor mutational burden (TMB) and tumor neoantigen burden (TNB) were significantly higher in responders than in non‐responders. Higher response rates and longer survival rates were observed in patients with higher TMB. Patients with higher ratios of CD8 to M2 macrophages had higher response rates and improved survival rates. Finally, the integrated analysis demonstrated that the combination of TMB and the ratio of CD8 T cells to M2 macrophages could further distinguish patients who benefitted from the treatment in both enrolled patients and public data.

**Conclusions:**

The findings of this study indicated that the combination of TMB and the ratio of CD8 T cells to M2 macrophages may be a potential biomarker for improving the recognition of NAC responders, thereby providing a basis for developing precision NAC regimens.

## Graphical abstract

In this study, we comprehensively analyzed the predictive efficacy of genomic and tumor microenvironment biomarkers and found that TMB and the ratio of CD8 T lymphocytes to M2 macrophages could predict the response to NAC in TNBC. More importantly, the combination of TMB and the ratio of CD8 T cells to M2 macrophages could further distinguish patients who benefitted from the treatment of NAC. These results were validated in the public data,which provide a basis for developing precision NAC regimens.

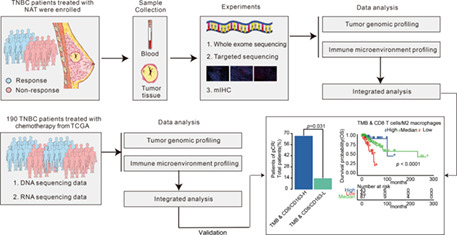

## 1 INTRODUCTION

Breast cancer is the most prevalent solid tumor, ranking first and fifth in terms of morbidity and mortality, respectively.^1^ Approximately 20% of breast cancer patients exhibit triple‐negative breast cancer (TNBC) characterized by a lack of estrogen receptors (ER), progesterone receptors (PR), and human epidermal growth factor receptor 2 (Her2) expression.^2^, ^3^, ^4^ Compared with other subtypes, TNBC exhibits a more aggressive biological behavior, earlier recurrence, and worse survival rate.^5^, ^6^, ^7^ Due to the lack of ER, PR, and Her2 expression, TNBC patients cannot benefit from anti‐HER2 therapy or endocrine therapy.^8^ Although neoadjuvant chemotherapy (NAC) is currently the most effective therapy for TNBC patients,^9^, ^10^, ^11^ only 35% of TNBC patients achieve a pathological complete response (pCR) after NAC.^12^ Therefore, biomarkers are urgently required to predict the efficacy and prognosis of NAC in TNBC patients.

Chemotherapy has the potential to promote the immunogenic death of tumor cells which can promote lymphocyte infiltration, and enhance the anti‐tumor immune response.^13^, ^14^ Tumor‐infiltrating lymphocytes (TILs), especially T lymphocytes, play an important role in anti‐tumor immunity.^15^ In the cancer‐immunity cycle, T lymphocytes kill tumor cells by recognizing neoantigens on the surface of tumor cells.^16^ Therefore, it is necessary to consider the effect of TILs on the response to chemotherapy. Neoantigens are associated with tumor mutational burden (TMB)^17^; patients with higher TMB can potentially form more neoantigens, thereby promoting anti‐tumor immune responses.^18^ TMB has been used to predict the efficacy of immune checkpoint inhibitors (ICIs) in various cancers, including TNBC.^19^, ^20^, ^21^, ^22^, ^23^, ^24^ In addition, previous studies have demonstrated that combination of TMB and immune gene expression profile (GEP) could further distinguish responders from non‐responders in TNBC patients treated with ICIs.^25^ However, whether TMB or a combination of TMB and TILs can predict the response to chemotherapy in TNBC is unclear.

In this study, we integrated the tumor genome and microenvironment to analyze the efficacy and clinical outcomes in TNBC treated with NAC and found that TMB and the ratio of CD8 T lymphocytes to macrophages could predict the response to NAC. More importantly, integration of TMB and the ratio of T lymphocytes to macrophages could further distinguish responders.

## 2 METHODS

### 2.1 Patient enrollment and data collection

Twenty‐one TNBC patients who underwent NAC from March 2019 to October 2020 at the Jiangsu Province Hospital were enrolled in the study. Tumor biopsies pre‐NAC treatment were collected for whole exome sequencing (WES), targeted sequencing, and multiplex immunohistochemistry (mIHC). The Residual Cancer Burden (RCB) system was conducted to access primary breast tumor and axillary lymph nodes status simultaneously,^26^, ^27^ and the Miller‐Payne (MP) system only compared the disparity of cell richness in the primary breast cancer lesions excluding lymph nodes.^28^ pCR was defined as no invasive cancer in the primary breast lesion and no cancer in the regional lymph nodes after NAC, but the in situ residuals in the breast was allowed. The pCR was equivalent to RCB0 and MP5 plus lymph node negativity. According to the MP and RCB systems,^29^ there were eight and six patients with MP5 grades and pCR, respectively (Table 1). In addition, due to the lack of multi‐omics data on neoadjuvant TNBC patients, publicly available data from 190 TNBC patients who had received adjuvant chemotherapy were downloaded as a validation cohort from The Cancer Genome Atlas (TCGA) in cBioPortal (Firehose Legacy). Patients with overall survival (OS) > 5 years were considered to benefit from the treatment. Detailed information on all patients is summarized in Table S1.

**TABLE 1:** Baseline characteristics and clinical response for enrolled patients

Patient characteristics (*N* = 21)	*N* (% or range)
Patient factors	
Age (mean), years	47 (28–61)
Gender	
Female	21 (100)
Male	0 (0)
Subtype	
Triple negative	21 (100)
Non‐triple negative	0 (0)
Tumor factors (before NAC)	
MR tumor size(cm)	
cT1	2 (9.5)
cT2	12 (57.1)
cT3	6 (28.6)
NA	1(4.8)
RCB system	
pCR (RCB0)	6 (28.6)
non‐pCR (RCBI‐III)	15 (71.4)
MP system	
MP5	8 (38.1)
MP1‐4	13 (61.9)

Abbreviations: cT, clinical T stage; MP system, Miller‐Payne system; RCB system, Residual Cancer Burden system.

### 2.2 Next‐generation sequencing (NGS) and data processing

DNA extraction and quantification: Formalin‐fixed paraffin‐embedded (FFPE) tumor tissue and matched peripheral blood samples were extracted using the GeneRead DNA FFPE Kit (QIAGEN, GER) and Mag‐Bind® Blood & Tissue DNA HDQ 96 Kit (OMEGA), respectively. Purified DNA was qualified using the Qubit dsDNA HS Assay Kit (Thermo Fisher Scientific). Sequencing libraries were prepared using Exome Plus Panel V1.0 (IDT, USA), and sequencing procedures were performed using the MGISEQ platform with 100 bp paired‐end reads. For WES, the medium depth of coverage was 234× for tumors and 231× for matched blood controls. For targeted sequencing, the medium depth of coverage was 1763× for tumors and 533× for matched blood controls.

Data processing: SOAPnuke (v1.5.6) was used to filter out results with a low quality and an N rate beyond 10%.^30^ The clean results were aligned with the human reference genome hg19 using the Burrows‐Wheeler Alignment tool (BWA, version 0.7.12) within the BWA‐mem algorithms.^31^ Alignment data conversion, sorting, and indexing were carried out using SAMtools (version 1.3).^32^ The duplicates were marked with SAMBLASTER (Version 0.1.22) to reduce biases.^33^

Mutation calling: VarScan (v2.4.1) and VarDict (1.7.0) were used to identify somatic mutations, including single nucleotide variants (SNVs) and insertions and deletions (indels).^34^, ^35^ The mutation was annotated using SnpEff (Version 4.3) software.^36^ The TMB was defined as the number of all nonsynonymous mutations and indels per mega base of the genome examined. MSIsensor (v0.2) was used to detect microsatellite instability (MSI) status.^37^ Then, the MSI value was recalculated and corrected using the in‐house tool.

### 2.3 Tumor neoantigen calculation

POLYSOLVER (v1.0) and Bwakit (v0.7.11) were used to type the human leukocyte antigen (HLA) of the tumor tissue and the matched peripheral blood samples.^38^ Then, HLA deletion was calculated with LOHHLA.^39^ All nonsynonymous mutations and indels were translated into 21‐mer peptide sequences using in‐house software centered on mutated amino acids. Then, the 21‐mer peptide was used to produce a 9‐ to 11‐mer peptide using a sliding window approach to predict the binding affinity of major histocompatibility complex (MHC) class I. Further, the binding strength of mutated peptides to HLA alleles was predicted using NetMHCpan (v3.0).^40^ If the predicted binding affinity with half‐maximum inhibitory concentration (IC50) was no larger than 500 nM, the peptide was selected, and several selected peptides generated from the same mutation were counted as one neoantigen. The tumor neoantigen burden (TNB) was defined as the number of putative neoantigens per mega base of the genome examined.

### 2.4 Evaluation of HLA loss of heterozygosity (HLA LOH) and intratumoral heterogeneity (ITH)

OptyType (v1.3.2) and POLYSOLVER (v1.0) were used to determine HLA typing of the tumor tissue and the matched peripheral blood samples.^38^, ^41^ The maintenance or loss of HLA was determined using LOHHLA.

ITH was calculated as previously described.^42^ Briefly, VarScan (v2.4.1) was used to detect mutations in tumor tissue samples.^34^ CNVkit (v0.8.1)^43^ was used to call copy number variants, and ascatNgs (v3.1.0) was used to assess tumor purity.^44^ The cancer cell fraction (CCF) of the mutations was calculated using PyClone (v0.13.0).^45^ The cut‐off values for ITH‐H and ITH‐L were defined as the median values of ITH.

### 2.5 Calculation of homologous recombination deficiency (HRD)‐score

The HRD‐score was calculated as previously described.^46^ Briefly, re‐alignment of reads was completed with GATK (3.8.0).^47^ Aligned bam files were analyzed with sequenza (3.0.0),^48^ and finally HRD‐score was detected using scarHRD (0.1.1).^49^

### 2.6 Immune infiltrate analysis

Transcriptome data of 189 TNBC patients from TCGA in cBioPortal (Firehose Legacy) were used to calculate the enrichment level of 11 major infiltrating lymphocytes, including CD8 T cells, dendritic cells (DCs), M2 macrophages, M1 macrophages, macrophages, regulatory T cells (Tregs), CD4 T cells, B cells, natural killer (NK) cells, neutrophils, and monocytes using the R package xCell which is a gene signatures‐based method learned from thousands of pure cell types from various sources,^50^ and the median values of different cell type were used as cut‐off value for dividing high and low.

### 2.7 mIHC staining

The mIHC was performed using a PE‐Opal 7‐color Automation IHC Kit (NEL821001KT, Perkin Elmer). FFPE blocks were heated at 65°C for 30 min. The slides were then dewaxed, rehydrated, and fixed using a Leica BOND RX auto Stainer (Leica Biosystems). Subsequently, the slides were stained with three markers: anti‐CD8 (790–4460, Ventana), anti‐CD163 (ab182422, Abcam), and anti‐Pan Keratin (790–2135, VENTANA), followed by incubation with horseradish peroxidase (HRP)‐conjugated secondary antibody and tyramide signal amplification (TSA). Finally, the slides were stained with 4′‐6′‐diamidino‐2‐phenylindole (DAPI) for 10 min at 1:10 dilution, and images were acquired using a Vectra 3.0 pathology imaging system microscope (PerkinElmer Inc.).

### 2.8 Statistical analyses

All statistical analyses were performed using the R software version 4.1.2. The Wilcoxon test was used to compare differences between groups. Fisher's exact tests were used to evaluate the categorical variables. Correlation analysis was conducted using the Pearson correlation analysis. Kaplan–Meier curve analysis was used to determine the significance of OS and progression‐free‐survival (PFS). Statistical significance was set at *p*‐value <0.05.

## 3 RESULTS

### 3.1 Overview of the study

**FIGURE 1:**
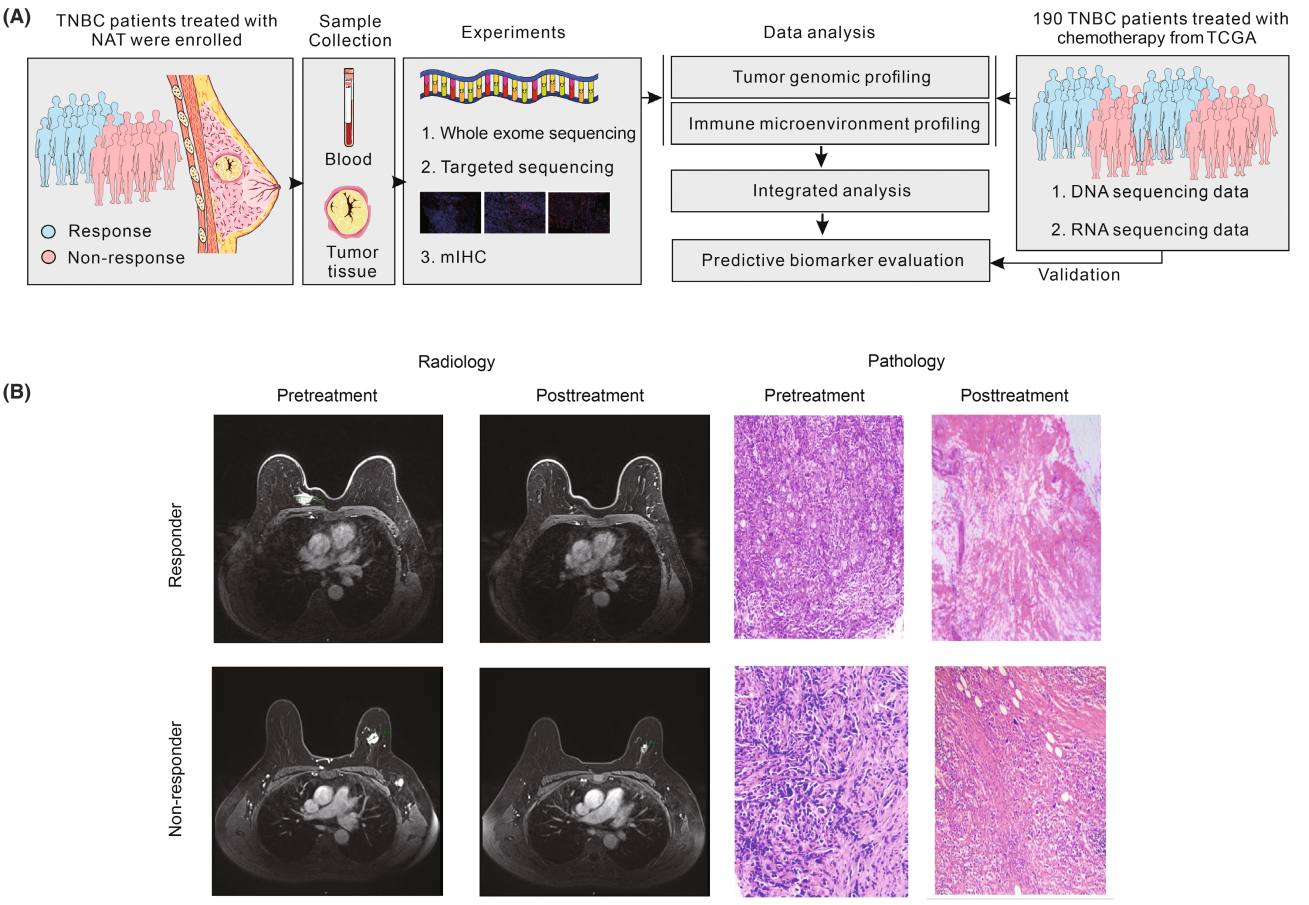
Overview of the study. (A) Sample collection and multi‐omics data processing. Our study included 21 enrolled TNBC patients and 190 TNBC patients from TCGA. Pretreatment biopsies of enrolled patients were collected and performed with WES, targeted‐panel sequencing and mIHC. Genomic data and transcriptome data of patients from TCGA were gained. And these multi‐omics data were processed to predicted the efficacy in TNBC. (B) Representative radiological and pathological images of responder and non‐responder.

As shown in Figure 1A, this study had three major aims. The first aim was to identify genomic biomarkers that could predict the response to NAC in TNBC patients. To accomplish this aim, biopsy samples of 21 TNBC patients were analyzed using WES and targeted gene sequencing. The correlation between genomic biomarkers and the response to NAC was analyzed, and further validated using public data. The second aim was to investigate the effect of tumor microenvironment on the response in TNBC patients. To achieve this, the predictive effect of various infiltrating lymphocytes related to the treatment response was analyzed using transcriptome data, and the results were further verified using mIHC data from enrolled patients. The last aim was to integrate genomic and tumor microenvironment biomarkers to predict the efficacy of NAC in TNBC. To this end, we first analyzed whether the integration could predict a response in the enrolled patients and then validated the results using public data. The representative radiological and pathological images are shown in Figure 1B. The response rates in the enrolled patients with pCR and MP5 grades were 28.6% and 38.1%, respectively (Table 1).

### 3.2 Genomic characteristics of tumor in enrolled TNBC patients

**FIGURE 2:**
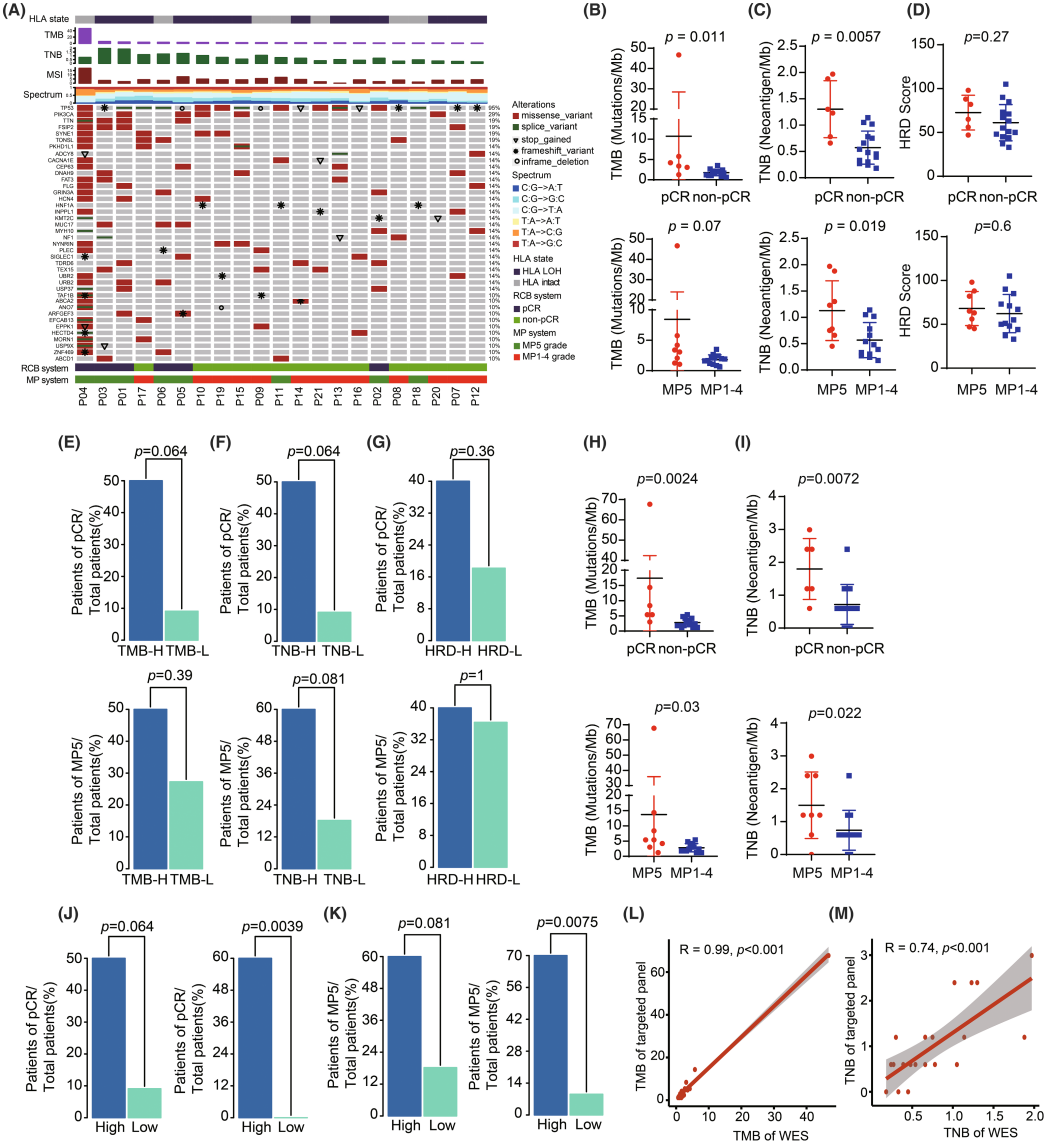
Genomic characteristics of tumor in enrolled TNBC patients. (A) The genomic landscape of TNBC patients treated with NAC. Top track is the state of HLA. Top histogram are the values of TMB, TNB and MSI of each patient. Mutation spectrum of each patient are shown under the value of MSI. Center heatmap is the distribution of non‐synonymous driver mutation events from patients; The bottom tracks are the response state of pCR and MP5 grade. (B–D) Comparison of TMB (B), TNB (C) and HRD score (D) with WES between responders and non‐responders. (E) Barplots of pCR rate and MP5 grade rate between TMB‐H group and TMB‐L group with WES. (F) Barplots of pCR rate and MP5 grade rate between TNB‐H group and TNB‐L group with WES. (G) Barplots of pCR rate and MP5 grade rate between HRD‐H group and HRD‐L group. (H) and (I) Comparison of TMB (H) and TNB (I) with targeted‐panel sequencing between responders and non‐responders. (J) Barplots of pCR rate and MP5 grade rate between TMB‐H group and TMB‐L group with targeted‐panel sequencing. (K) Barplots of pCR rate and MP5 grade rate between TNB‐H group and TNB‐L group with targeted‐panel sequencing. (L) and (M) Correlation of TMB (L) and TNB (M) between WES and targeted‐panel sequencing.

To explore biomarkers predicting efficacy in TNBC treated with NAC, WES was performed on 21 enrolled TNBC patients. The genomic landscape is shown in Figure 2A. Consistent with previous studies,^51^ the most frequently mutated genes in TNBC patients were *TP53* (95%), *PIK3CA* (29%), *TTN* (19%), *FSIP2* (19%), *SYNE1* (19%), *TONSL* (19%), *PKHD1L1* (14%), *ADCY8* (14%), *CACNA1E* (14%), and *CEP63* (14%) (Figure 2A and Table S2). One patient had a relatively high TMB and MSI. The detailed characteristics are shown in Table S1.

To further investigate genomic biomarkers associated with response to NAC, we compared the responders and non‐responders, and found that TMB and TNB were significantly higher in pCR patients than in non‐pCR patients (Figure 2B,C). TMB and TNB were higher in patients with MP5 grade than in patients with MP1‐4 grades (Figure 2B,C). However, there was no difference in ITH and tumor volume between responders and non‐responders (Figure S1A,B). A previous study demonstrated that HRD could predict the response to chemotherapy in TNBC. In our study, we found that the HRD‐score was higher in the responders than in the non‐responders; however, there was no significant difference (Figure 2D). Further, we analyzed the response rates in these patients and found that the rate of pCR was correlated with TMB and TNB (Figure 2E,F). However, the rate of MP5 grade was discovered to be correlated with TNB, but not with TMB (Figure 2E,F). There was no correlation between response rates and HRD, ITH, tumor volume or HLA LOH (Figure 2G and Figure S1C–E).

As targeted panel sequencing was superior to WES in terms of cost, time, and availability of biopsies, we also performed panel sequencing on all samples (Table S3). Consistently, TMB and TNB were significantly higher in responders than in non‐responders (Figure 2H and I). The response rates of the pCR and MP5 grade were higher in patients with TMB‐H or TNB‐H (Figure 2J,K), respectively. Subsequently, we compared the correlation between TMB and TNB values calculated using WES and targeted panel sequencing and found that TMB and TNB values detected by panel sequencing were significantly correlated with those detected by WES. (Figure 2L,M).

**FIGURE 3:**
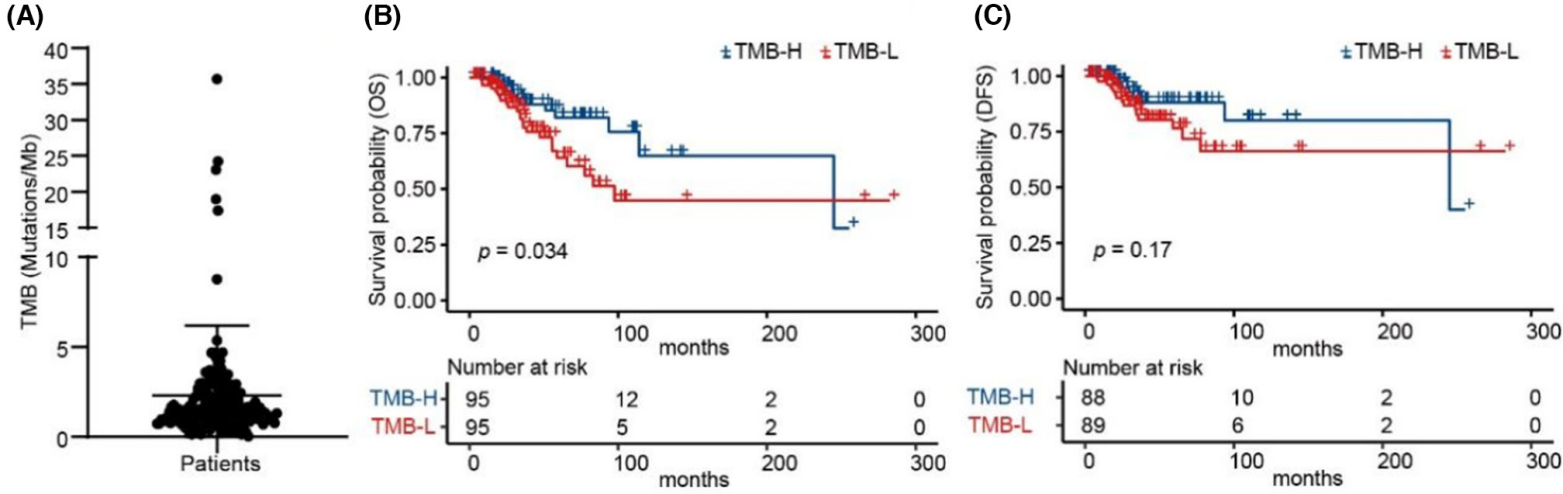
Patients of TNBC with TMB‐H had a longer survival. (A) Distribution of TMB from TNBC patients in TCGA. (B) and (C) Kaplan–Meier curves of OS (B) and DFS (C) comparing TMB‐H with TMB‐L from TNBC patients in TCGA.

To verify whether TMB could predict the efficacy in TNBC patients, the genomic data of 190 TNBC patients obtained from TCGA was further analyzed. As shown in Figure 3A, the highest TMB of these patients was 35.7, the lowest TMB was 0.033, and the median TMB was 1.45. We analyzed the relationship between TMB and prognosis and found that OS was longer in TMB‐H patients than in TMB‐L patients (Figure 3B,C). The results demonstrated that patients of TNBC with TMB‐H had a longer survival rate.

### 3.3 Integration of CD8 T lymphocytes and M2 macrophages further distinguishes patients who benefitted from the treatment

**FIGURE 4:**
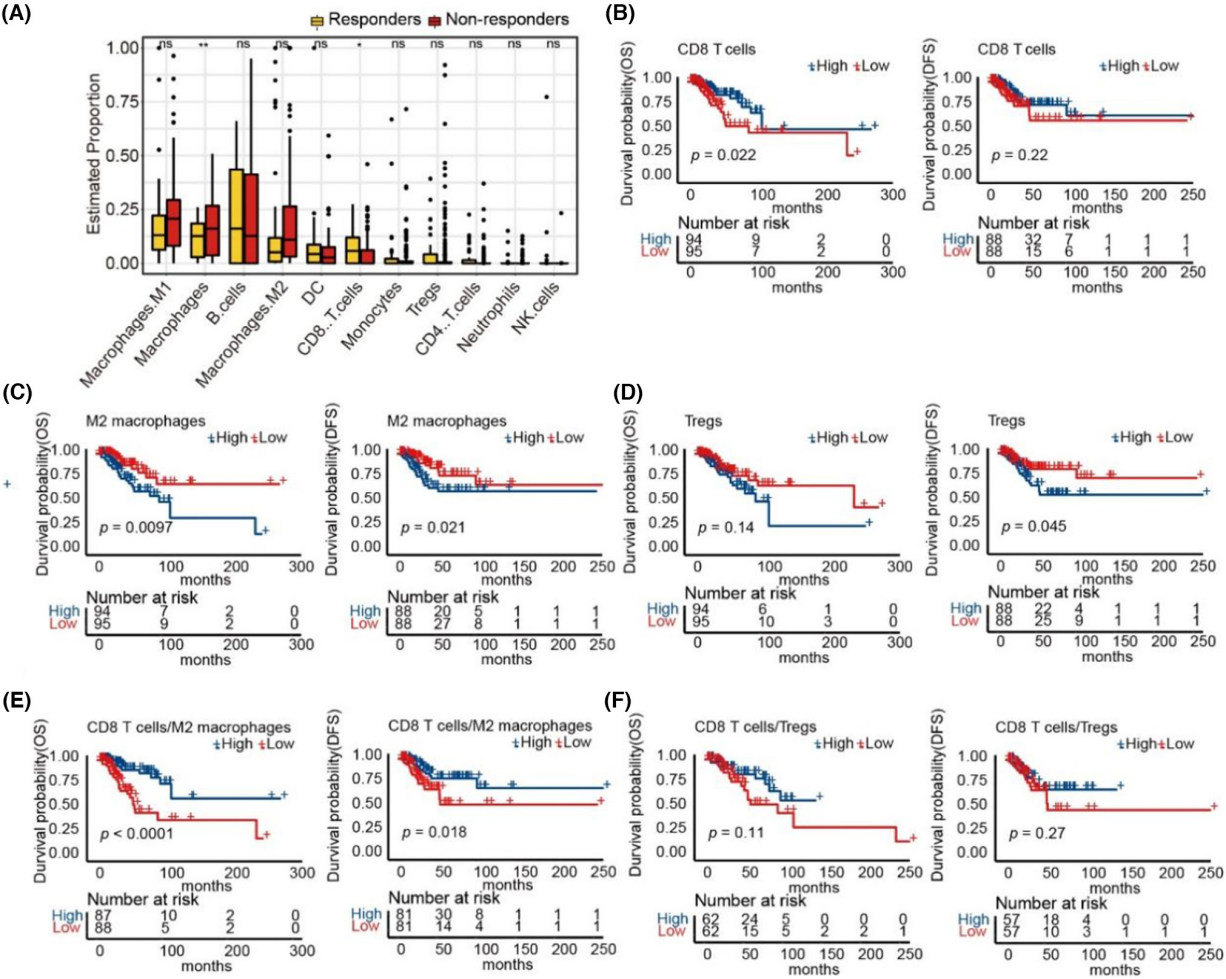
Integration of CD8 T lymphocytes and M2 macrophages can further distinguish patients who benefitted from the treatment. (A) Proportion of 11 major TILs from TNBC patients in TCGA. (B) Kaplan–Meier curves of OS and DFS comparing CD8 T cells‐H with CD8 T cells ‐L from TNBC patients in TCGA. (C) Kaplan–Meier curves of OS and DFS comparing M2 macrophages‐H with M2 macrophages‐L from TNBC patients in TCGA. (D) Kaplan–Meier curves of OS and DFS comparing Tregs‐H with Tregs‐L from TNBC patients in TCGA. (E) Kaplan–Meier curves of OS and DFS comparing CD8 T cells/M2 macrophages‐H with CD8 T cells/M2 macrophages‐L from TNBC patients in TCGA. (F) Kaplan–Meier curves of OS and DFS comparing CD8 T cells/Tregs‐H with CD8 T cells/Tregs‐L from TNBC patients in TCGA.

The tumor microenvironment, especially TILs, plays a critical role in the treatment of TNBC. To investigate whether TILs could predict the efficacy in TNBC, the abundance of 11 major TILs, including CD8 T cells, DCs, M2 macrophages, M1 macrophages, macrophages, Tregs, CD4 T cells, B cells, NK cells, neutrophils, and monocytes were analyzed. As shown in Figure 4A, macrophages were significantly lower, while CD8 T cells were significantly higher in patients with OS >5 years than others. Furthermore, we analyzed the survival of patients with different TILs and found that patients with higher CD8 T lymphocytes, lower M2 macrophages, and lower Tregs survived longer (Figure 4B–D). However, B cells, CD4 T cells, DCs, macrophages, M1 macrophages, NK cells, neutrophils, and monocytes did not predict the response to chemotherapy (Figure S2A–H).

Since the presence of CD8 T lymphocytes, M2 macrophages, and Tregs could predict the efficacy of chemotherapy in TNBC, we hypothesized that the integration of these infiltrating cells may further distinguish patients who benefitted from the treatment. As shown in Figure 4E, the DFS and OS were longer in patients with a high ratio of CD8 T lymphocytes to M2 macrophages than in those with a low ratio of CD8 T lymphocytes to M2 macrophages. However, there was no difference in the ratio of CD8 T lymphocytes to Tregs (Figure 4F).

### 3.4 The response rate to NAC was higher in patients with a higher ratio of CD8 to CD163

**FIGURE 5:**
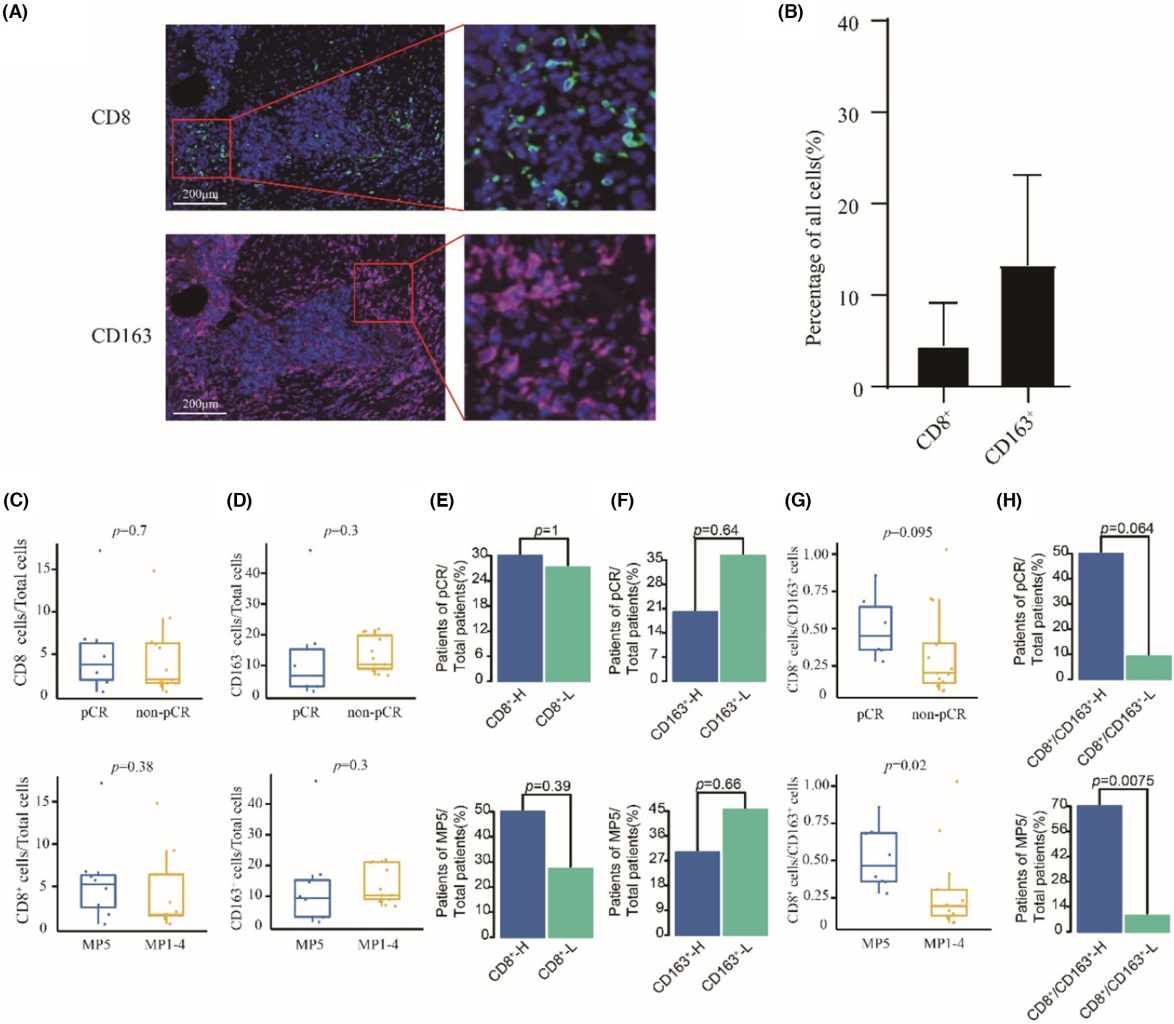
The response rate was higher in patients with high ratio of CD8 to CD163. (A) Representative images from mIHC analysis of CD8 T cell marker (CD8: blue) and M2 macrophage marker (CD163: red). (B) Distribution of CD8 and CD163 from 21 TNBC patients. (C–D) Comparison of CD8 (C) and CD163 (D) between responders and non‐responders. (E) Barplots of pCR rate and MP5 grade rate between CD8‐H group and CD8‐L group. (F) Barplots of pCR rate and MP5 grade rate between CD163‐H group and CD163‐L group. (G) Comparison of CD8/CD163 between responders and non‐responders. (H) Barplots of pCR rate and MP5 grade rate between CD8/CD163H group and CD8/CD163‐L group.

To further verify the predictive efficacy of the ratio of CD8 T cells to M2 macrophages in TNBC patients treated with NAC, we performed mIHC with CD8 and CD163 surface markers in enrolled patients (Figure 5A). In these patients, the proportions of CD8 T lymphocytes and CD163 macrophages were 4.75% and 13.45%, respectively (Figure 5B). Compared with non‐responders, responders had higher CD8 T lymphocytes and lower CD163 macrophages (Figure 5C,D), which was consistent with the above results. The predictive value of CD8 T lymphocytes and CD163 macrophages was then explored, and there was no significant difference (Figure 5E,F). However, the ratio of CD8 T lymphocytes to CD163 macrophages was higher in responders than in non‐responders (Figure 5G). Furthermore, the response rate of patients with a higher ratio of CD8 T lymphocytes to CD163 macrophages was also higher (Figure 5H).

### 3.5 Integration of TMB and ratio of CD8 T lymphocytes to M2 macrophages could further predict the efficacy in TNBC

**FIGURE 6:**
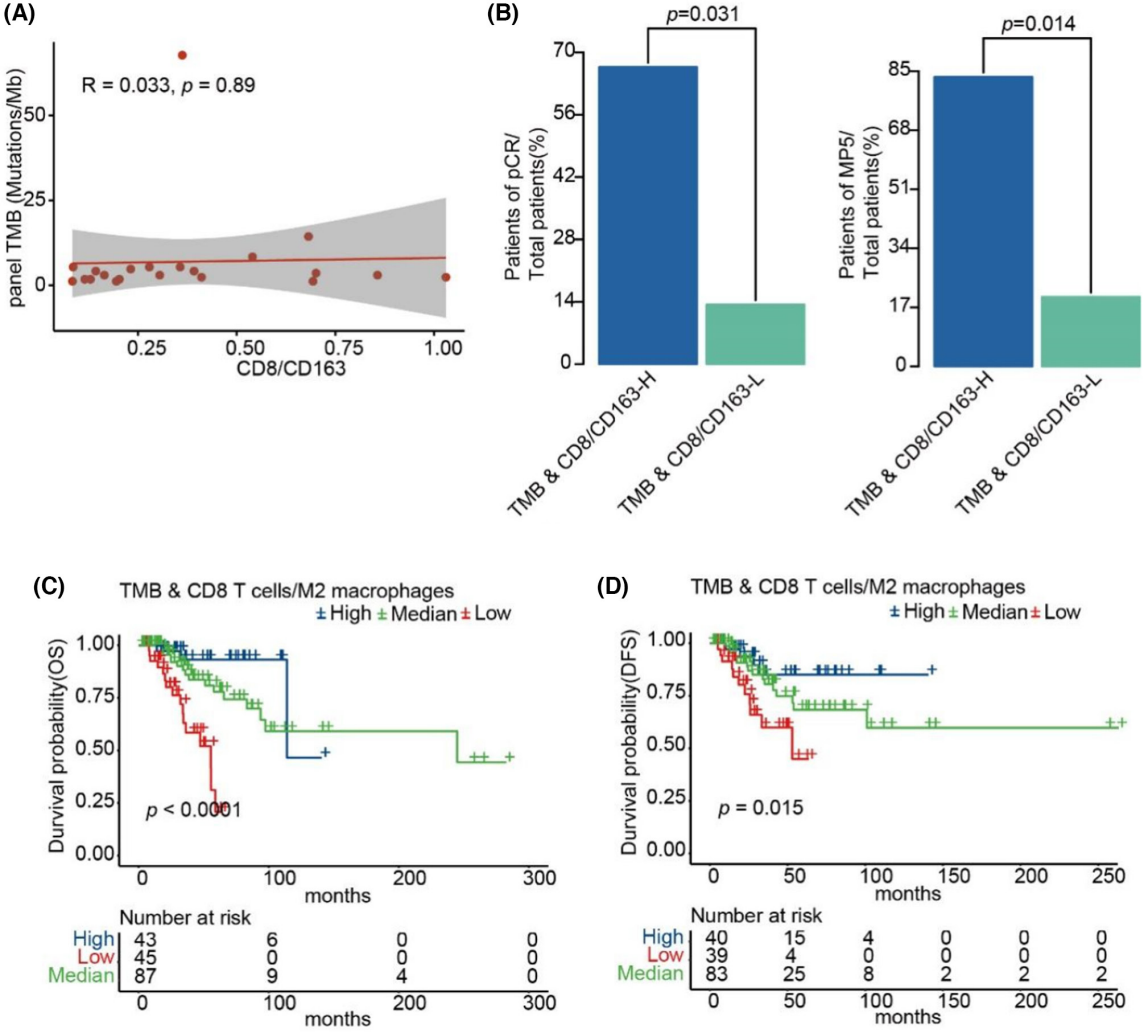
Integration of TMB and the ratio of CD8 T lymphocytes to M2 macrophages could further predict the efficacy in TNBC. (A) Correlation of TMB of targeted‐panel sequencing and CD8/CD163. (B) Barplots of pCR rate and MP5 grade rate between TMB & CD8/CD163‐H group and TMB & CD8/CD163‐L group. (C) and (D) Kaplan–Meier curves of OS and DFS comparing TMB & CD8 T cells/M2 Macrophages‐H with TMB & CD8 T cells/M2 Macrophages‐L from TNBC patients in TCGA.

To examine whether integrating tumor genomic and immune microenvironment features had improved prediction of the efficacy for TNBC, we performed integrated analysis by combining both TMB and the ratio of CD8 T lymphocytes to M2 macrophages. As shown in Figure 6A, there was no significant correlation between TMB and the ratio of CD8 T lymphocytes to M2 macrophages. We then integrated TMB and the ratio of CD8 T lymphocytes to M2 macrophages to predict the efficacy and found that patients with a high TMB and a high ratio of CD8 T lymphocytes to M2 macrophages had a greater response rate than others (Figure 6B). The findings were further validated by using the multi‐omics data of 190 TNBC patients of TCGA. As shown in Figure 6C,D, patients in TCGA with a high TMB and a high ratio of CD8 T lymphocytes to M2 macrophages had the longest survival rates, while patients with a low TMB and low ratio of CD8 T lymphocytes to M2 macrophages had the shortest survival.

## 4 DISCUSSION

Currently, NAC is the standard treatment for TNBC; however, the beneficiaries are limited, indicating that biomarkers are urgently needed to predict its efficacy.^52^ In this study, we comprehensively analyzed the predictive efficacy of genomic and tumor microenvironment biomarkers and found that TMB and the ratio of CD8 T lymphocytes to M2 macrophages could predict the response to NAC in TNBC. More importantly, patients with both high TMB and a high ratio of CD8 T lymphocytes to M2 macrophages had longer survival rates.

TMB has been reported to be related to the efficacy of chemotherapy in various cancer types, such as lung, gastric, and colorectal cancers.^53^, ^54^, ^55^, ^56^ However, in TNBC, the effect of TMB on the efficacy of chemotherapy is unclear. A previous study revealed that patients with higher TMB in TNBC treated with chemotherapy had a higher survival rate.^57^ However, another retrospective study demonstrated that TMB was higher in the short DFS group than that in the long group.^58^ Because the study only included 14 non‐pCR patients, the conclusions of this study need to be confirmed further. In our study, we not only confirmed that patients with a high TMB had a higher response rate in our self‐enrolled cohort, but also confirmed this conclusion in public data from TCGA, which indicated that patients with a high TMB are more likely to benefit from chemotherapy treatment in TNBC. In addition, to improve the clinical application of TMB, we also performed panel sequencing on the self‐enrolled patients and found that the results of panel sequencing were highly correlated with the results of WES.

TILs play an important role in the treatment of TNBC,^59^, ^60^, ^61^ especially CD8 T lymphocytes, which are important anti‐tumor immune effector cells, and their infiltration can affect the state of the tumor immune microenvironment. The greater the infiltration of CD8 T lymphocytes, the stronger the anti‐tumor immunity. M2 macrophages trigger an immunosuppressive microenvironment and inhibit the anti‐tumor immune response within TNBC tumors.^62^ In addition, several studies have revealed that the combination of the CD8 T cells and M2 macrophages was associated with clinical benefits.^63^, ^64^, ^65^ Similar results were observed in our study. Compared with non‐pCR patients, pCR patients had more CD8 T lymphocytes and fewer M2 macrophages. The ratio of CD8 T lymphocytes to macrophages was higher in pCR patients than in non‐PCR patients, and patients with a higher ratio of CD8 T lymphocytes to macrophages had a higher response rate, which indicated that TNBC patients with immune‐activated tumor microenvironments could benefit more from chemotherapy.

Tumors have complex ecosystems, and a single biomarker has a limited ability to predict treatment efficacy.^66^, ^67^ Previous studies have shown that integrating multiple genomic markers or integrating genomic and transcriptome features can further distinguish responders from non‐responders.^25^, ^68^, ^69^ In TNBC, patients with homologous recombination defects and immune activation may benefit more from chemotherapy treatment.^51^ In our study, we integrated genomic biomarkers and tumor microenvironment biomarkers and found that patients with high TMB and a high ratio of CD8 T lymphocytes to M2 macrophages had a higher response rate to chemotherapy in TNBC.

The relative enrichment levels of infiltrating lymphocytes from TCGA were inferred based on expression profiles of related gene. However, there were several limitations with transcriptome‐based assessment of the infiltrating lymphocytes. Prediction accuracy is susceptible to the differences of cellular RNA content. And the calculation of infiltrating lymphocytes particularly depends on the related marker genes.^50^ Therefore, it is necessary to consider the infiltrating lymphocytes with mIHC in future studies. In addition, there were only response data, and no survival data in the enrolled patients. In fact, it is very complicated to specifically predict whether patients benefit from treatment. Therefore, it is necessary to obtain data of different dimensions for comprehensive evaluation. Finally, due to the small sample size and lack of multi‐omics data from public datasets of neoadjuvant TNBC patients, publicly available data from 190 TNBC patients who had received adjuvant chemotherapy were used as validation cohort. Therefore, further neoadjuvant treatment studies in TNBC patients were needed to validate our results.

In summary, we have shown that TMB and the ratio of CD8 T lymphocytes to M2 macrophages could serve as a single biomarker to predict the efficacy in TNBC patients treated with NAC. Further, the integration of TMB and the ratio of CD8 to CD163 could further distinguish patients who benefitted from the treatment. The results may help clinicians make personalized and precise treatment decisions.

## AUTHOR CONTRIBUTIONS

**Yanhui Zhu:** Data curation (equal); formal analysis (equal); funding acquisition (equal); methodology (equal); writing – original draft (equal). **Hongfei Zhang:** Data curation (equal); formal analysis (equal); methodology (equal); writing – original draft (equal). **Chaohu Pan:** Data curation (equal); formal analysis (equal); methodology (equal); writing – original draft (equal). **Gao He:** Formal analysis (equal); writing – original draft (equal). **Xiaoli Cui:** Formal analysis (equal); writing – original draft (equal). **Xiafei Yu:** Supervision (equal); writing – review and editing (equal). **Xiaoqiang Zhang:** Supervision (equal); writing – review and editing (equal). **Dongfang Wu:** Supervision (equal); writing – review and editing (equal). **Junzhe Yang:** Supervision (equal); writing – review and editing (equal). **Xian Wu:** Supervision (equal); writing – review and editing (equal). **Haitao Luo:** Conceptualization (equal); project administration (equal); supervision (equal); writing – review and editing (equal). **xiaoan liu:** Conceptualization (equal); funding acquisition (equal); project administration (equal); supervision (equal); writing – review and editing (equal).

## FUNDING INFORMATION

This work was funded by the National Natural Science Foundation of China (82072931 and 82002805).

## CONFLICT OF INTERESTS

Author Chaohu Pan, Xiaoli Cui, Dongfang Wu and Haitao Luo were employed by the company YuceBio Technology Co. The remaining authors declare that they have no competing interests.

## ETHICS APPROVAL AND CONSENT TO PARTICIPATE

This study was approved by the Institutional Ethics Committees at The First Affiliated Hospital of Nanjing Medical University. Tumor and blood samples collection were conducted in accordance with the Declaration of Helsinki. Written informed consent was obtained from all participants. The ethical approval number was 2022‐SR‐005.

## CONSENT TO PUBLICATION

Not applicable.

## Supporting information


Figure S1

Figure S2

Table S1

Table S2

Table S3

## Data Availability

The raw data of TNBC patients generated in this study are available from the corresponding author on reasonable request. Clinical information of all patients is summarized in Table S1. Public data of 190 TNBC patients were downloaded from TCGA in cBioPortal (Firehose Legacy) (https://www.cbioportal.org).
