# Supplementary figures and images for: Integrated tumor genomic and immune microenvironment analysis identifies predictive biomarkers associated with the efficacy of neoadjuvant therapy for triple‐negative breast cancer
Source: Cancer Med. 2022 Oct 21;12(5):5846–58. doi: 10.1002/cam4.5372 (PMC10028167; doi:10.1002/cam4.5372)

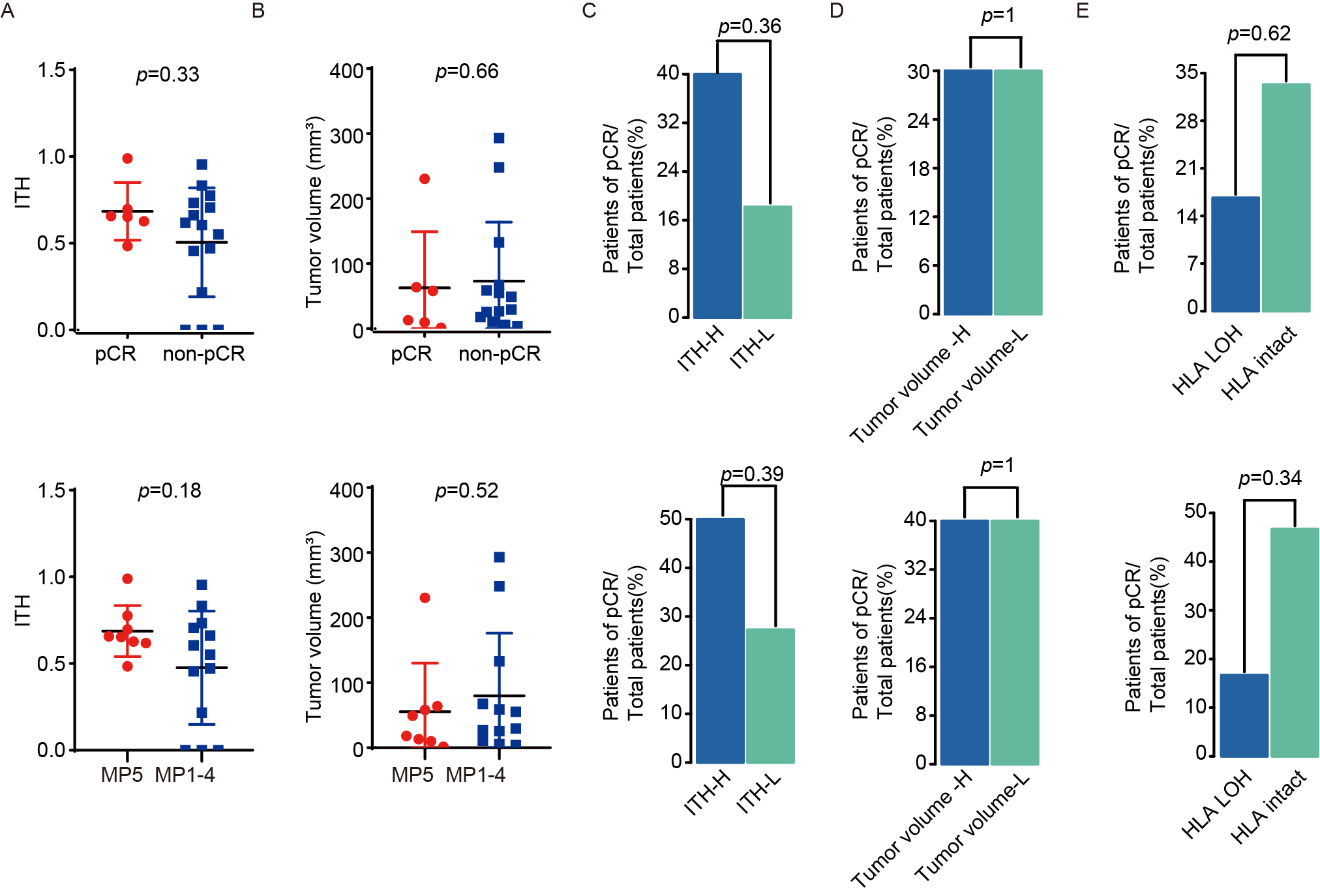

Supplement: Supplementary file 1 — Figure S1 [file CAM4-12-5846-s001.jpg]

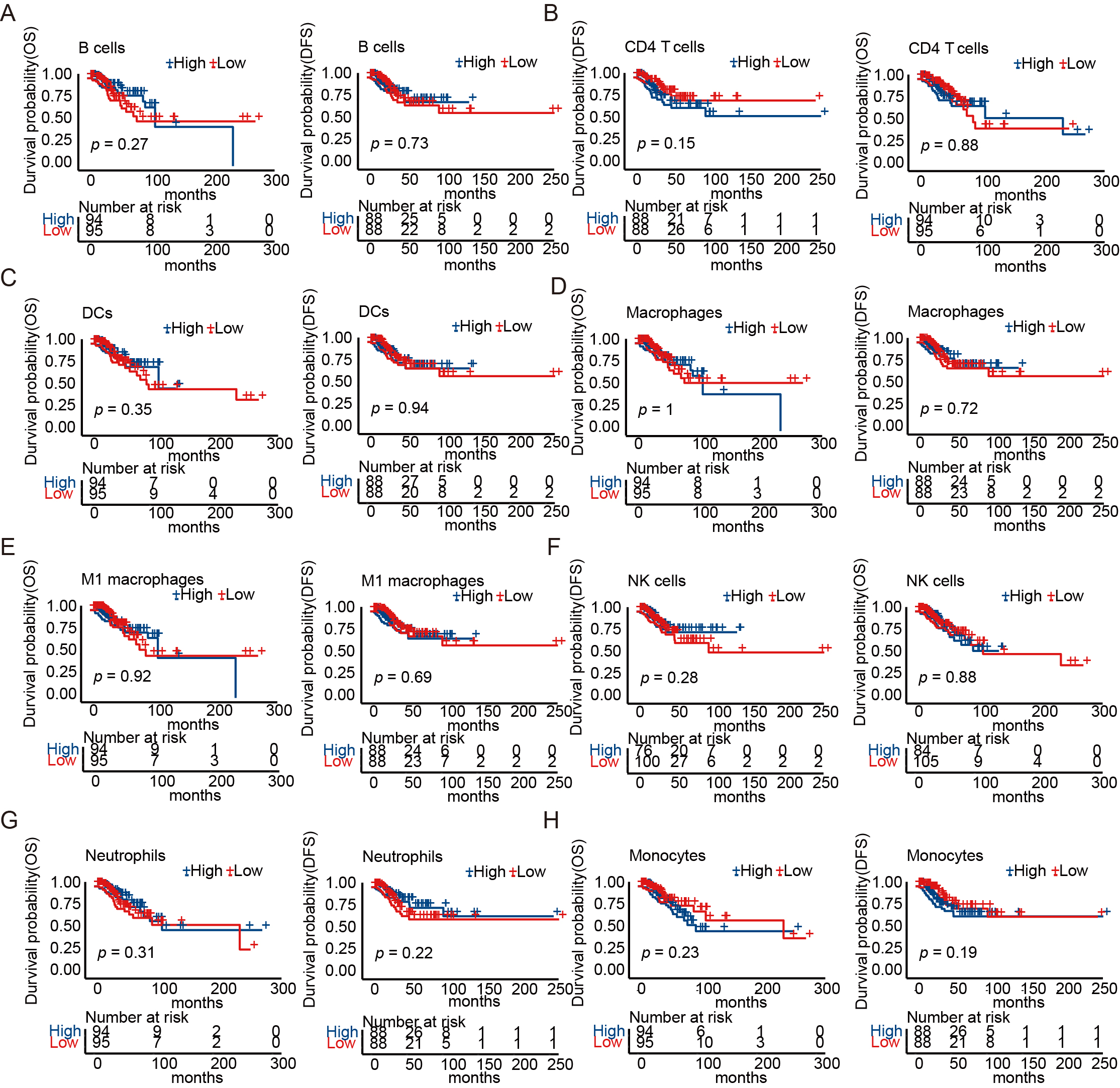

Supplement: Supplementary file 2 — Figure S2 [file CAM4-12-5846-s003.jpg]
